# Demographic Histories, Isolation and Social Factors as Determinants of the Genetic Structure of Alpine Linguistic Groups

**DOI:** 10.1371/journal.pone.0081704

**Published:** 2013-12-02

**Authors:** Valentina Coia, Marco Capocasa, Paolo Anagnostou, Vincenzo Pascali, Francesca Scarnicci, Ilaria Boschi, Cinzia Battaggia, Federica Crivellaro, Gianmarco Ferri, Milena Alù, Francesca Brisighelli, George B. J. Busby, Cristian Capelli, Frank Maixner, Giovanna Cipollini, Pier Paolo Viazzo, Albert Zink, Giovanni Destro Bisol

**Affiliations:** 1 Accademia Europea di Bolzano (EURAC), Istituto per le Mummie e l'Iceman, Bolzano, Italy; 2 Dipartimento Biologia e Biotecnologie “Charles Darwin”, Sapienza Università di Roma, Rome, Italy; 3 Istituto Italiano di Antropologia, Rome, Italy; 4 Dipartimento Biologia Ambientale, Sapienza Università di Roma, Rome, Italy; 5 Istituto di Medicina Legale e delle Assicurazioni, Università Cattolica di Roma, Rome, Italy; 6 Sezione di Antropologia, Museo Nazionale Preistorico Etnografico “Luigi Pigorini”, Rome, Italy; 7 Dipartimento Integrato di Servizi Diagnostici e di Laboratorio e di Medicina Legale, Università di Modena e Reggio Emilia, Modena, Italy; 8 Department of Zoology, University of Oxford, Oxford, United Kingdom; 9 Dipartimento Culture, Politica e Società-Sezione Scienze Antropologiche, Università degli Studi di Torino, Turin, Italy; University of Utah, United States of America

## Abstract

Great European mountain ranges have acted as barriers to gene flow for resident populations since prehistory and have offered a place for the settlement of small, and sometimes culturally diverse, communities. Therefore, the human groups that have settled in these areas are worth exploring as an important potential source of diversity in the genetic structure of European populations. In this study, we present new high resolution data concerning Y chromosomal variation in three distinct Alpine ethno-linguistic groups, Italian, Ladin and German. Combining unpublished and literature data on Y chromosome and mitochondrial variation, we were able to detect different genetic patterns. In fact, within and among population diversity values observed vary across linguistic groups, with German and Italian speakers at the two extremes, and seem to reflect their different demographic histories. Using simulations we inferred that the joint effect of continued genetic isolation and reduced founding group size may explain the apportionment of genetic diversity observed in all groups. Extending the analysis to other continental populations, we observed that the genetic differentiation of Ladins and German speakers from Europeans is comparable or even greater to that observed for well known outliers like Sardinian and Basques. Finally, we found that in south Tyroleans, the social practice of *Geschlossener Hof*, a hereditary norm which might have favored male dispersal, coincides with a significant intra-group diversity for mtDNA but not for Y chromosome, a genetic pattern which is opposite to those expected among patrilocal populations. Together with previous evidence regarding the possible effects of “local ethnicity” on the genetic structure of German speakers that have settled in the eastern Italian Alps, this finding suggests that taking socio-cultural factors into account together with geographical variables and linguistic diversity may help unveil some yet to be understood aspects of the genetic structure of European populations.

## Introduction

A considerable body of evidence shows that geographic distance is a good predictor of the genetic structure of European populations. A southeast-northwest cline, possibly associated with the Pleistocene settlement of the continent and the Neolithic demic diffusion from the Fertile Crescent [1,2] (but see 3), has been initially highlighted for classic genetic markers [1] and later corroborated by the analysis of Y chromosome and autosomal polymorphisms [4,5,6]. One exception to this scenario, however, is that no clear evidence of clinal variation has been observed for mitochondrial DNA, which is supposedly a consequence of the higher female compared to male migration associated with the prevalence of patrilocality [7,8,9]. Finns, Sardinians, Basques and European Jewish provide important departures from this pattern, a finding which is currently explained by bottlenecks and/or their reduced genetic exchange with other European populations [10,11,12,13,14,15]. A potential but yet to be well explored source of diversity in the European genetic landscape is represented by groups that have settled in mountainous environments. In particular, great mountain ranges, such as the Alps, Pyrenees and Carpath, may have not only acted as barriers to gene flow for resident populations, but have possibly, since prehistory, also offered a place for the settlement of small, and sometimes culturally diverse, communities. 

The Alps are one of the broadest mountain ranges of Europe, with a longitudinal extension of approximately 1,200 kilometers. They cover eight different countries and over 100 peaks of over 4000 m a.s.l. There is a substantial consensus among archeologists regarding the notion that many alpine areas had already been inhabited in the Paleolithic [16,17], with a more intense peopling starting from the Neolithic [18,19]. However, occupation of the upper valleys remained scattered and small in number until a more systematic process of colonization and demographic expansion began in the late Middle Ages [20]. Another key passage concerning the demographic history of the Alps is represented by the “break-up of isolates”. In fact, a dramatic decline of endogamy began in the first half of the 20th century due to an increase in individual mobility and the depopulation of the mountain areas thanks to socio-cultural changes linked to industrialization [21,22].

At present, Alpine populations can be considered as a mosaic of groups that are separated by physical and cultural boundaries, whose remarkable cultural diversity is clearly demonstrated by the presence of minorities that speak Franco-Provençals, Occitans, French, German, Ladin, Friulian and Sloven languages [23,24]. From a bio-anthropological point of view, they offer a unique opportunity to study the impact of geographical, demographic and cultural factors on genetic structure [25]. Such a target requires the simultaneous investigation of distinct linguistic groups and, ideally, the analysis of genetic systems with different modes of evolution and transmission. Unfortunately, the population genetic studies that have been carried out so far are scanty and most of them only focused on a limited number of populations or single groups [26,27,28,29,30].

In this study, we present new high resolution data on Y chromosomal variation in three distinct Alpine ethno-linguistic groups, Italian, Ladin and German. Combined with data on Y chromosome and mitochondrial variation taken from our previous research work and the literature, these results are used to answer four questions: (i) how is genetic diversity patterned in alpine ethno-linguistic groups?; (ii) what micro-evolutionary forces might have shaped their genetic structure?; (iii) how do the observed patterns compare with what has been noticed in other European groups, in particular with well known genetic outliers and other groups settled in great mountain ranges?; (iv) are there factors, other than geography and language, that should be taken into account when studying the genetic structure of European mountain populations?

## The Populations under Study

**Figure 1:**
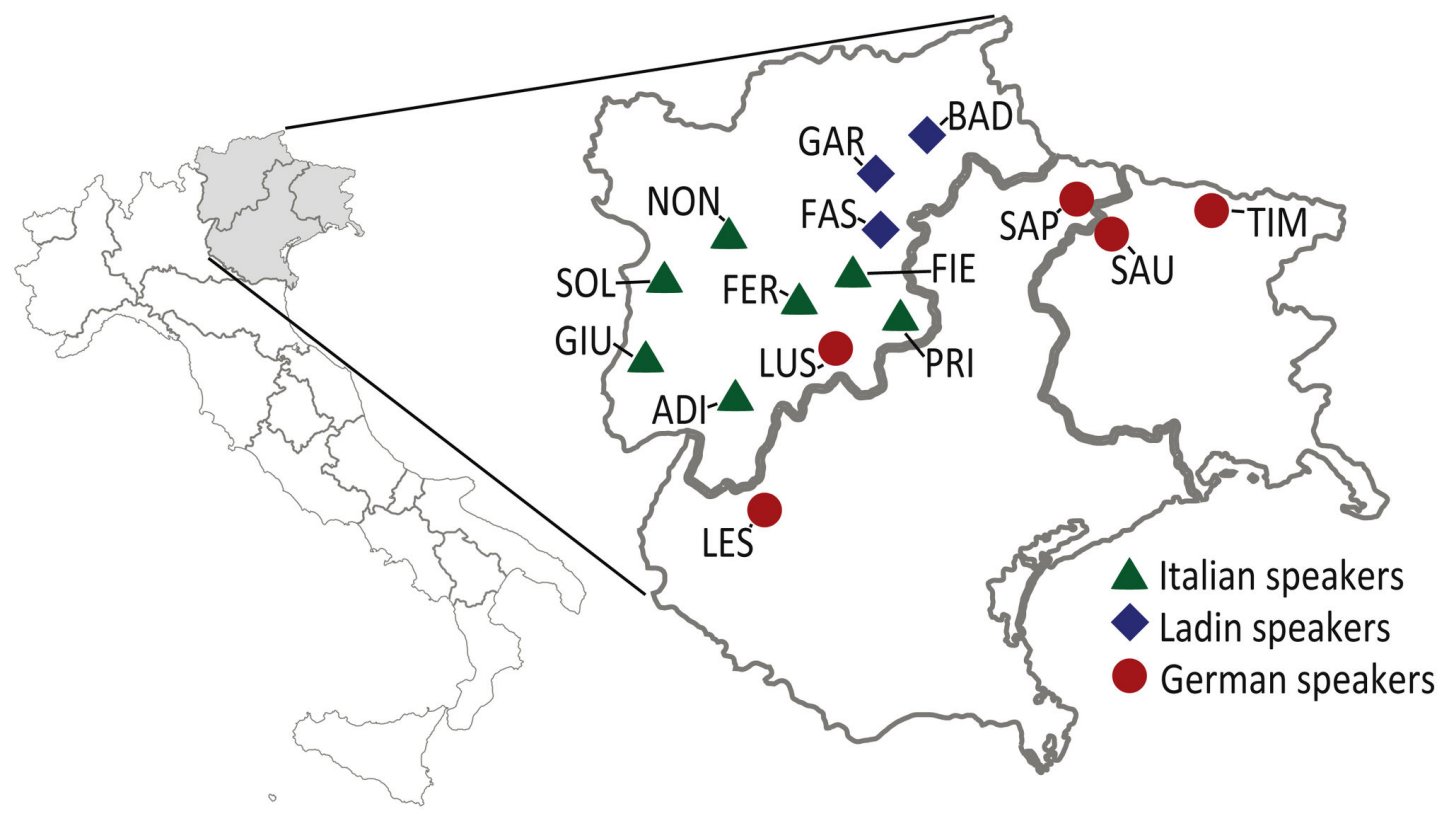
Geographic location of the populations under study (see table 1 for population acronyms).

Our study is primarily based on unpublished Y chromosome data (17 Short Tandem Repeats, STRs, and 50 Single Nucleotide Polymorphisms, SNPs) from 610 unrelated individuals belonging to 15 populations from the Eastern Italian Alps (Trentino-Alto Adige, Veneto and Friuli regions; see Table 1 and Figure 1).

**Table 1:** Populations included in the present survey.

**Population (region)**	**Abbreviation**	**Sample size**	**Language**	Census size^*^
Adige (Trentino)	ADI	56	Romance (Italian)	166394
Badia (South Tyrol)	BAD	44	Romance (Ladin)	10644^†^
Fassa (Trentino)	FAS	47	Romance (Ladin)	9894^†^
Fersina (Trentino)	FER	26	Romance (Italian)	2575
Fiemme (Trentino)	FIE	41	Romance (Italian)	18990
Gardena (South Tyrol)	GAR	51	Romance (Ladin)	10198^†^
Giudicarie (Trentino)	GIU	51	Romance (Italian)	36282
Lessinia (Veneto)	LES	24	German	13455^§^
Luserna (Trentino)	LUS	25	German	286
Non (Trentino)	NON	48	Romance (Italian)	37832
Primiero (Trentino)	PRI	41	Romance (Italian)	9959
Sappada (Veneto)	SAP	38	German	1307
Sauris (Friuli)	SAU	29	German	429
Sole (Trentino)	SOL	65	Romance (Italian)	15235
Timau (Friuli)	TIM	24	German	500

* ISTAT (2011) (http://demo.istat.it)

^†^ This value refers to Ladin speaking communities only [23]

^§^ This value refers to Cimbrian speaking communities only [23]

Ten populations belong to the main Romance language [Italians (Adige, Fersina, Fiemme, Giudicarie, Non, Primiero and Sole valleys); Ladins (Fassa, Badia, and Gardena valleys)], five to the German-linguistic isolates [two Cimbri groups (from Luserna and the Lessinia area); the communities of Sappada, Sauris and Timau]. 

Ladins are thought to be related to pre-Indo-European speaking tribes who probably represent the most ancient settlers of the Alps [31]. The Dolomitic Ladins are the remnant of a wider group that started settling in a broader territory in 1000 AD. As for the Ladins, the other Romance speaking groups of Italians are thought to be linked to the most ancient peopling of the area [31]. Finally, the ethno-linguistic Germanic islands of the Eastern Alps are in continuity with nuclei that migrated from Bavaria, Carinthia and Tyrol in the late Middle Ages, a process driven by the landed aristocracy and the monasteries with the objective of a more intensive exploitation of marginal territories [20]. 

The dataset was integrated with an extensive search of literature data on unilinear transmitted markers [32] relative to populations living in the Alps or in other European mountain ranges (Pyrenees) (see Table S1). 

## Material and Methods

### Sampling and ethic statements

Buccal swabs were collected in apparently healthy and unrelated donors selected according to the place of birth of the sampled individual and of their parents and grandparents. The procedure and informed consent were reviewed and approved by the “*Comitato Etico per la Sperimentazione con l’Essere Umano*” of the University of Trento (samples from Trentino), “South Tyrolean Ethics Committee” (samples from Alto Adige, POLYS project) and the institutional review board of the Istituto Italiano di Antropologia (samples from Veneto and Friuli). All participants provided written informed consent to participate in this study.

### Laboratory analyses

The DNA was extracted using the ‘Nucleic Acid Isolation System’ by the QuickGene-810 instrument following the standard protocols for blood and swab samples (FUJIFILM) or using a modified “salting-out” procedure. 

The 17 Y-chromosomal short tandem repeats (STRs) included in the AmpFlSTR Yfiler Amplification Kit (AB Applied Biosystems; DYS19, DYS389I, DYS389II, DYS390, DYS391, DYS392, DYS393, DYS385ab, DYS437, DYS438, DYS439, DYS448, DYS456, DYS458, DYS635 and GATA H4.1) were typed in all samples (with the exclusion of 59 samples from Non and Sole valleys belonging to the R-M269* lineage which had been previously published [3]). PCR products were analyzed by capillary electrophoresis in an ABI 3100 Genetic analyzer (Applied Biosystem, Foster City, CA). Fifty Y-specific unique-event polymorphisms were examined in hierarchical order (M17, M102, M153, M170, M172, M173, M201, M222, M223, M224, M241, M253, M26, M267, M269, M280, M282, M304, M319, M35, M410, M423, M438, M45, M47, M521, M67, M78, M89, M9, M92, P37.2, S116, S127, S139, S144, S145, S167, S21, S28, S29, SRY2627, V12, V13, V148, V19, V22, V27, V32, V65). Firstly, all samples were tested by one basal multiplex (MY1) following the protocol reported in Onofri et al. [33] with the addition of UEPs M269, M17, M201, M267, M282 and M304. Afterwards, all the samples derived for the M269 mutation (T>C), M35 (C>G), M170 (A>C), and M172 (A>C) were further analyzed using the specific multiplex for haplogroups R1b*, E*, I* and J2* , respectively ([3,34] and Brisighelli F and Capelli C, personal communication). The protocol includes first PCR amplification reactions by using the Qiagen Multiplex PCR kit with the conditions specified by the producer [35] and subsequent purification by enzymatic method (ExoSAP; [36]). The purified products were then used for a single-base extension reactions by the SNAPShot method (Applied Biosystems Carlsbad, CA). 

Phylogenetic relationships between markers and nomenclature follow the International Society of Genetic Genealogy (April 2013, Ver 8.43), (http://www.isogg.org/tree/). The population data obtained were submitted to the Anthro-Digit database (http://www.isita-org.com/Anthro-Digit/data.htm).

### Statistical analysis

Unless otherwise stated, statistical analyses were performed using 15 STRs, having excluded the duplicated DYS385 loci. The level of intra-population genetic variation was analyzed through the calculation of haplotype diversity (HD) and the number of different haplotypes (H). Multi-Dimensional Scaling of Fst genetic distances based on Y chromosome STRs (Reynolds’ distances, [37]) and a Principal Component Analysis plot based on haplogroup frequencies were obtained using SPSS software (release 16.0.1 for windows, SPSS Inc.). We partitioned genetic variance at different hierarchical levels of population subdivision according to language groups (Italian, Ladin and German) by means of a molecular analysis of variance (AMOVA). In this analysis, we also used mitochondrial DNA literature data (HVR1, 333 bp from 16033 to 16365; see Table S2) [32]. All parameters of intra and inter-population genetic diversity were calculated using the Arlequin software (version 3.5.1.2, [38]).

We used a coalescent based simulation approach in order to evaluate whether the observed values of within-group genetic diversity may be attributed solely to the size of the founding group (see Tofanelli et al. [39] for a review of simulation methods for uniparental markers). We separated Italians into two sub-groups, western and eastern, according to their different current census size and previous mtDNA evidence [40]. Adige valley and Cimbrian populations were not considered to be part of the simulations because of the difficulties and uncertainties in modeling their evolutionary history. Based on current historical records, we designed two different topologies, one for the German-speaking island group and one for the two Italian sub-groups and Ladin speaking group. In both topologies (see Figure S1) three sub-populations split from a large source population at a certain time (T1) which were identified as Central-Western Europe but which differ in splitting times (32-40 generations for German speaking islands and 90-110 generation for all the other groups). According to Bramanti et al. [41], effective population sizes for source and sink populations were set as 1/10 of census size. Growth rate for the source population was set at 0.0018 from 1800 to 300 generations ago, and increased to 0.022 from then to the present day [42]. The growth rate for the sink populations was set as half of the highest value of the source. A symmetrical gene flow between source and sink was allowed (0.005-0.01), while admixture between sink populations was allowed to vary between 0.01-0.02 and 0.02-0.03. We simulated 10K random genealogies for the Y chromosome (15 STRs) using the mutation rate estimates of Ballantyne et al. [43] assuming a generation time of 25 years. For each scenario, we randomly sampled 50 individuals from each sink population and analyzed within-group diversity for each simulation using Arlequin 3.5 [38].

## Results and Discussion

### Patterns of genetic diversity in the linguistic groups of the Italian Alps

The Eastern Italian Alps embrace an important portion of the ethno linguistic diversity of the alpine arch, encompassing Romance (including Ladins and Italians) and German speakers. Their genetic characterization highlights a high level of diversity not only among single populations, but also within linguistic groups, a pattern which is likely to be due to a complex interplay of demographic histories and isolation determined by environmental and cultural factors. 

**Figure 2:**
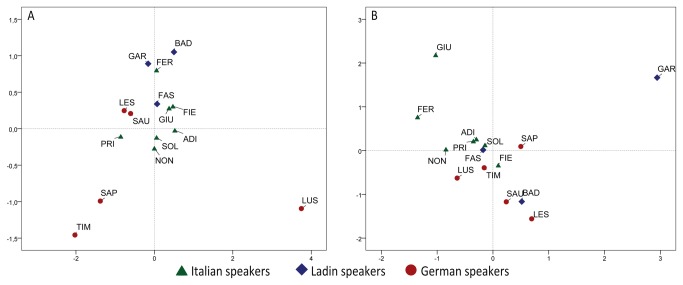
Plots of the genetic relations among populations under study. (a) Multi-Dimensional scaling plot of Fst genetic distances (15 STRs; stress value=0.128); (b) Principal Component Analysis plot based on haplogroup frequencies. First component (x axis) and second component (y axis) explain 16.96% and 13.95% of total variance, respectively. Acronyms are given in Table 1.

The extent of diversity among Alpine populations is shown by the plots based on STR and SNP data (Figure 2A and 2B). The spatial relationships among populations differ between the two plots, with the SNP-based patterns probably mirroring more ancient population relationships due to their slower evolutionary rate. However, with both data-type populations under study are well separated and no linguistic structure of genetic diversity is detectable. This latter feature may be appreciated in a quantitative way by an AMOVA performed among linguistic groups, which produced low values of intergroup variation (from 0.007 to 0.020; see Table S3). 

To gain further insights into the genetic diversity occurring within each linguistic group, we went one step further by focusing on their genetic structure. The Italian speaking group was found to be the most genetically homogeneous. Within group variation (0.04, p<0.05) is lower than in other Alpine groups and geographically distant European populations, but higher than observed among Northern Italian populations (Table S4-S5). Furthermore, they show high haplotype diversity values, with the highest observed in the Adige valley (0.997 ± 0.004 ; see Table S6). R1b S28*, a haplogroup found at high frequencies in most Alpine groups, is the most frequent in all populations (from 17 to 45%), the only exception being the Primiero, where G-M201 prevails (~49%, see Table S7). This pattern may be explained in two, not mutually exclusive, ways. Italian speaking populations have, since historical times constituted the most numerous ethno-linguistic group in the Eastern Alps, and they did not suffer from any historically documented bottleneck. Their present census values are comparable or higher than those of the other two groups under study (see Table 1). Furthermore, having settled in zones which are characterized by wider valleys, lower altitudes (from 200 to 1022 m a.s.l.) and more accessible mountain passes, they have probably been less geographically isolated than other groups (e.g. Ladins). Finally, the Adige river has provided a supplementary communication route, favoring population movements and interactions [44].

The genetic differentiation among Ladin valleys noticed in the plots is supported by other analyses of STR haplotype distribution. Their intra-group variation (0.075, p<0.05) is similar to what has been found in geographically distant European populations (6 populations, distances ranging from 366 to 2520 km, 0.074 p<0.05; see Table S4-S5) but much higher than what has been found in a set of Northern Italian populations (6 populations, distances ranging from 57 to 396 km, 0.006 p>0.05; see Table S4-S5) . Another likely effect of genetic drift may be seen in the intra-population diversity values (HD), which are lower than those observed in Italian speaking communities and in most European populations (Table S6). This is particularly evident for the communities from the Gardena and Badia valleys (South-Tyrol), which, correspondingly, depart more evidently from the main central group in the genetic distance plot (Figure 2A). Signatures of intra-group diversity are also provided by a phylogeographic approach. A further signal of the high within-group diversity is given by the finding that the prevalent haplogroup in the Fassa and Badia communities (S28*-R1b*) and Gardena valley (S-145 R1b*; Table S7) do not coincide. These two lineages of the main S116-R1b* haplogroup show a quite distinct continental distribution, with the former reaching its highest frequencies in south-central Europe (with frequencies peaks in France, northern Italy and the Alps), and S145-R1b found mainly in the north-Atlantic Europe [3,45]. On the whole, our results support the definition of Ladins as “small genetically isolated populations (subject to strong genetic drift), having a predominantly European ancestry” [27]. However, it should be noted that the inclusion of a third population (Fassa Valley) and the higher resolution of Y chromosome genotyping make our inferences more robust. The genetic signatures we observed may be an echo of the processes of fragmentation and/or assimilation of Ladin communities, first by Latin groups starting from the 15th century b.C, and then by German-speaking people (Gardena and Badia valleys) from the end of the 4^th^ century, and the consequent reduction in their settlement area and demographic size [46]. Moreover, the considerable altitude of the Ladin valleys (from 1120 to 1345 m a.s.l.) might have further increased a reciprocal isolation among fragmented Ladin communities [31]. 

The German speaking populations show the most marked signatures of genetic drift. As predicted by the outlying positions of Sappada, Timau and Luserna in the plot of genetic distances, the intra-group variation is very high (0.240, p<0.05), around two times higher than that found for geographically distant European populations. Moreover, the haplotype diversity values in these populations are the lowest of the the dataset, with the exception of Lessinia (see Table S6). Different haplogroups prevail in Sappada (E1b-V13 63%) and Timau (R1a-M17 56%), and different R1b subhaplogroups in Sauris (S139 34%), Lessinia (S116 17%) and Luserna (M269 84%). The considerable differentiation among German-speaking populations may be also seen as a consequence of their demographic history. In fact, they are in continuity with small founding groups [47] which settled in the present day location in Medieval times. Furthermore, as we have recently proposed [30], a relative reciprocal isolation could have occurred even among the linguistically closely related communities of Sappada, Timau, and Sauris as a result of “local ethnicity”. In this condition, the members of each community tend to identify their ancestry with their own village rather than considering themselves as part of the same ethnic group, similarly to what occurs in other alpine regions [48].

The genetic differentiation between the two Cimbri populations of Luserna and Lessinia deserves further discussion. Both these communities derive from Bavarian populations that colonized a vast territory of the Eastern Italian Alps starting from 1053 AD (Veneto; [49]) to 1216 AD (Trentino; [44]). Luserna is genetically very distant from all the other Alpine populations (average Fst=0.328; see Table S6) and shows a strikingly low intra-population diversity (0.483±0.119). Paternal lineages are represented mostly by the R1b-M269* (frequency of 84%), with six different STR haplotypes associated with only one founder surname. Lessinia shows different, if not opposite, features. The average genetic distances from other populations (Fst=0.097; see Table S6) is less than one third compared to Luserna, while HD is close to the highest values of our dataset (0.978±0.019; Table S6). The prevalent haplogroup, R1b-M269*, accounts for only one third of the total, the rest represented by different lineages (G-M201, I1-M253, M410-J2A and K-M9), which are associated with twenty-three different surnames. The demographic history of the Luserna and Lessinia communities may help explain their differentiation. Luserna was founded by few families which moved from Lavarone, the first known Cimbrian settlement in the territory of Trentino [44]. This could have led to a strong founder effect in this community, a hypothesis supported by a previous study of mtDNA polymorphisms [40]. Moreover, Luserna is located on a high plateau (1,333 m a.s.l.) and is quite isolated from the surrounding areas. By contrast, Lessinia, a more extensive area with reliefs of low altitude (Giazza, 758 m a.s.l.), and has been colonized since the XIII century AD through several migration waves of small groups of settlers for more than one century. From the XV century AD, this community opened to, and probably admixed with, Italian neighboring groups [49].

On the whole, our genetic characterization indicates three main genetic patterns. Italian speaking populations show slightly higher level of within-group diversity than observed among distant European populations. The strongest signals of departure from the European genetic background can be seen among German speaking populations, while the intra-group and intra-population diversity level of Ladins fall between the former two groups. These signals seem to reflect the different demographic history of the three groups and their genetic isolation due to the mountainous environment (for all groups) and use of different languages from their neighbors (Ladins and German speakers). Nonetheless, the fact that the Y chromosome is a single locus transmitted by father to sons means that our inference needs further support from other genetic systems with a diverse mode of inheritance. Therefore, we thought it would be useful to repeat the analysis of intra and inter-population diversity with maternally transmitted mitochondrial DNA polymorphisms (hypervariable region 1) [27,30,40]. Despite some minor differences regarding Sauris and Ladins from the Gardena valley (both show an outlying position in the mtDNA MDS plot and the latter a lower rank for haplotype diversity), mtDNA and Y chromosome patterns substantially match (see Figures S2 - S4 and Table S3).

**Figure 3:**
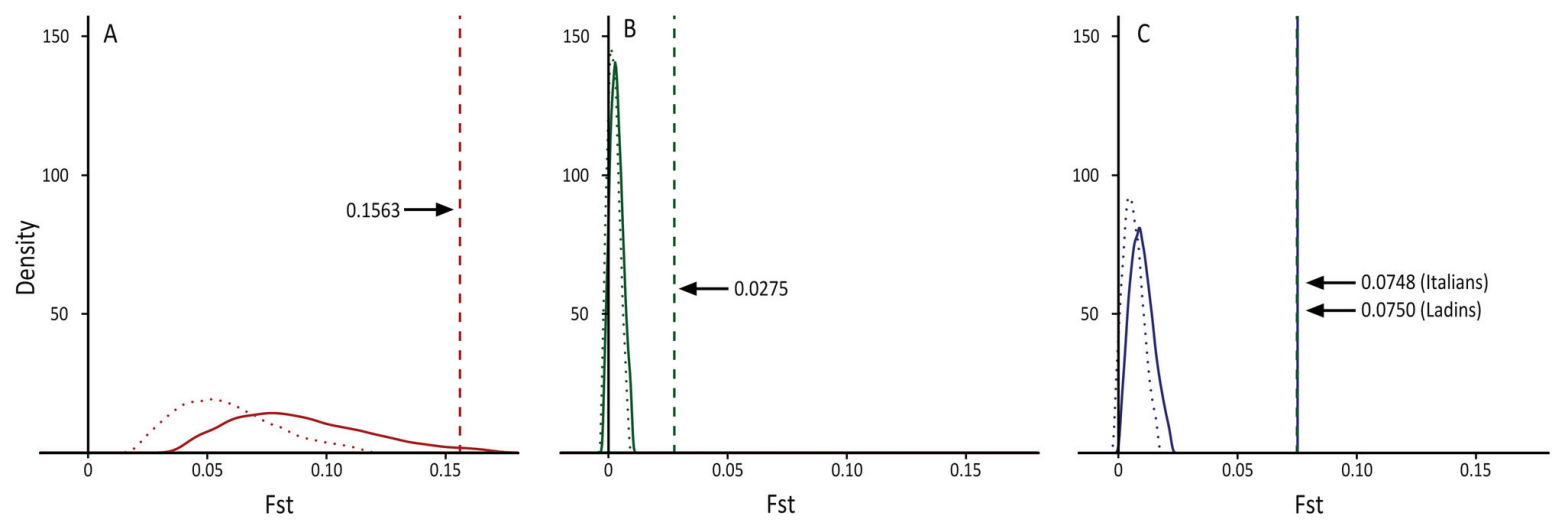
Posterior densities of Fst genetic distances for the micro-evolutionary scenarios. Distributions of Fst values obtained by coalescent simulations (see Materials and Methods), with vertical lines representing observed values of within group diversity: (A) German speaking islands; (B) Italians (Non, Sole and Giudicarie valleys); (C) Ladins (continuous) and Italians (dashed; Fiemme, Fersina and Primiero valleys).

As discussed above, the intensity of the genetic signals observed in the Alpine linguistic groups seems to comply with what is to be expected for isolated population groups characterized by a different demographic profile. Therefore, a cause effect relationship between these two conditions and the different patterns of genetic diversity is worth taking into consideration. However, it did not escape our attention that such intensity seems to be inversely correlated with the supposed size of the founding groups, reflecting present census values (see Table S8). We then decided to test the alternative hypothesis that our observations could be the result of differences in the long–term effective size among groups, without any substantial effect of genetic isolation. To this purpose, we carried out coalescent simulations for all of our linguistic groups, with comparable levels of gene flow to those expected for non isolated groups. The distributions obtained (Figure 3) are incompatible (Ladins and Italians) or only marginally compatible (German speakers) with the observed Fst values. A scenario combining the effects of founding group size and continued genetic isolation seems, therefore, to provide the best explanation for the observed level of within-group differentiation detected in both geographic and geographic/linguistic isolates.

### The Alpine linguistic groups in the European genetic background

**Figure 4:**
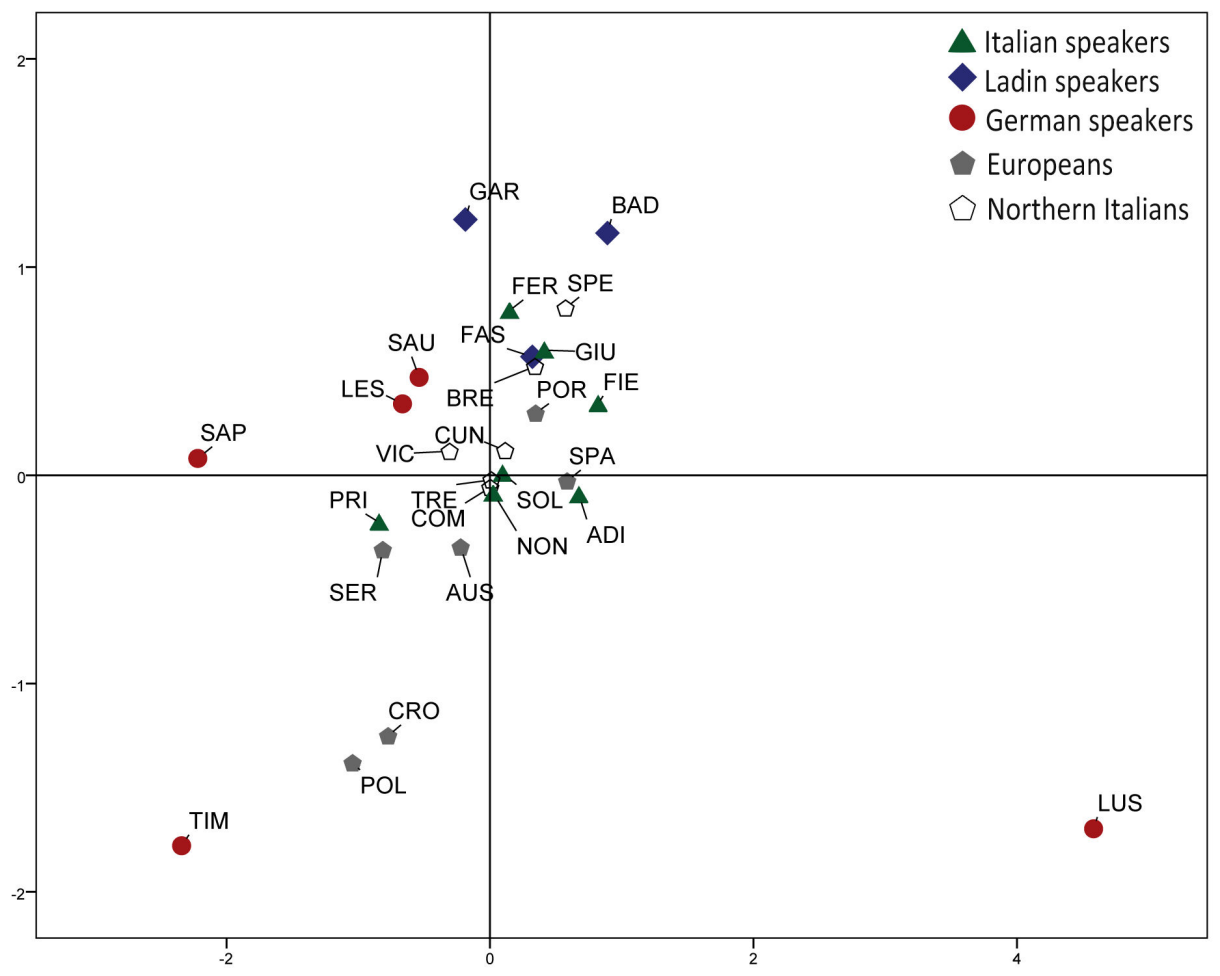
Multi-Dimensional Scaling plot of Fst genetic distances between Alpine and European populations. Plot based on 15 Y chromosome STRs (stress value=0.141). Population acronyms are given in Table 1 and Table S4.

**Figure 5:**
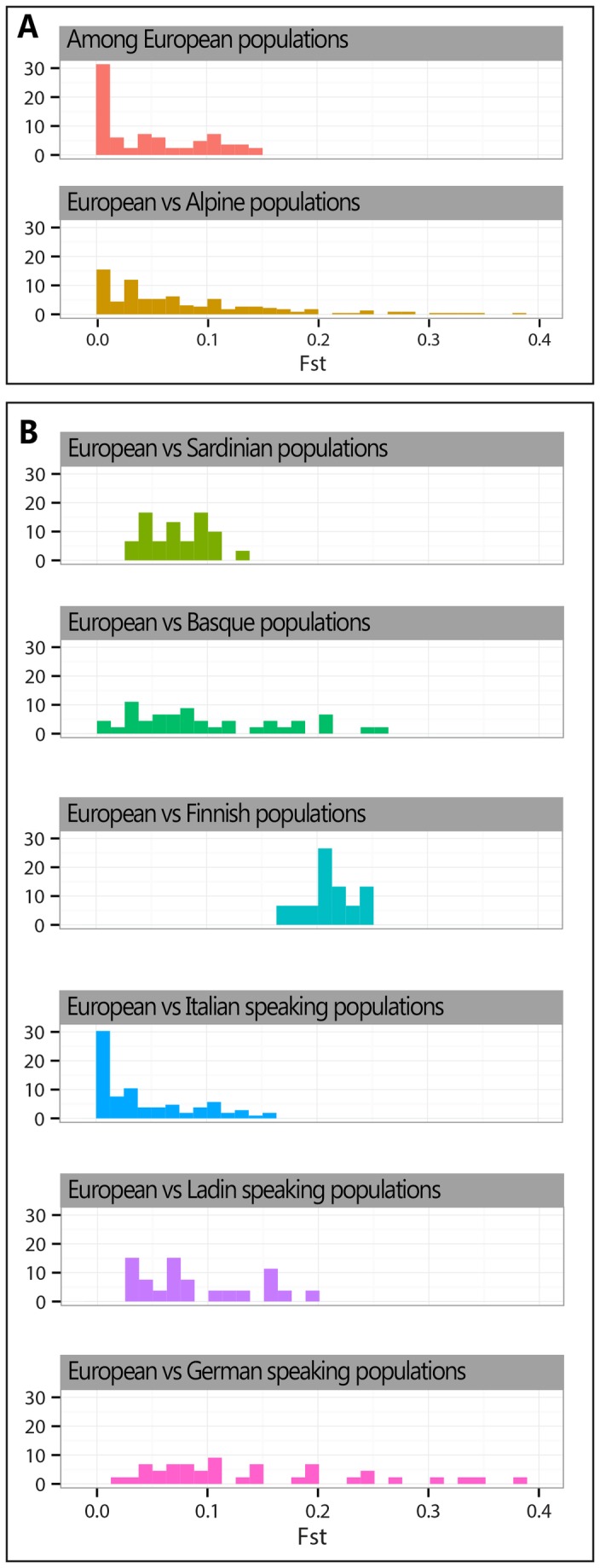
Distribution of Fst genetic distances among Alpine, Sardinian, Basque, Finn and European populations. Frame A shows the Fst genetic distances distributions among European populations and between European and Alpine populations (see Table S4; Italy is represented only by La Spezia). Frame B shows the Fst genetic distances between some European outliers (Sardinians, Basques and Finns), Alpine groups and European open populations.

The genetic distinctiveness of Alpine populations can be better appreciated contextualizing our results into the body of knowledge regarding European populations. A first comparison is to be made with open populations, to see whether group under study actually depart from the continental genetic structure. As shown by Roewer et al. [50], the distribution of Y chromosome variation at the continental level complies with an isolation by distance model. By contrast, the historical stratification and complexity of the peopling processes occurred in the Eastern Alps does not predict any simple relation between genetic structure and geographic distances. Accordingly, the correlation between geographic and Y chromosomal genetic distances is statistically insignificant (Spearman’s rho correlation value, R^2^= 0.61, p=0.99). Coherently, the alpine populations are widely dispersed in the MDS plot of Y-STR genetic distances despite their geographic proximity (Figure 4), with Sappada, Timau and Luserna behaving as outliers. The histogram plot (Figure 5A) based on 15 Y-STR loci highlights the substantial departure of Alpine populations from open continental groups (Austria, Croatia, Italy, Poland, Portugal, Serbia and Spain; see Table S4). In fact, average and median value of genetic distances between Alpine and open populations (0.095; 0.078) are substantially greater than between the latter (0.061; 0.061). 

Using the same approach (Figure 5B), we observed that the genetic differentiation of Ladins and German speakers from Europeans is comparable or even greater to that observed for well known continental outliers (see Table S4). In fact, the average value of Ladins (0.092) is higher than Sardinians (0.078), whereas their median Fst is slightly lower (0.075 vs 0.088). The signal is even stronger for the German speakers, whose average (0.144) exceeds that of Basques (0.121), whereas the two median values are rather close (0.111 vs 0.121) and 14.3% of Fst is above the upper bound of the range of genetic distances between Europeans and Basques. However, all these values are lower than those obtained for Finns (average 0.209; median 0.208) who are known to have undergone severe bottlenecks and further local episodes of drift [13].

As the final step of our study, we further extended our dataset by including other populations that have settled in great mountain range systems, from the Pyrenees (5) [51] and from South Tyrol (3) [27]. The results of the AMOVA (Table 2 and Table S9) show that Y chromosome intra-group variation within human groups that have settled in mountainous environments is relatively high and statistically significant, the South Tyroleans being the only exception (see below). Not unexpectedly, this is in sharp contrast with the low and insignificant diversity observed among open populations settled on plains at comparable geographic distances (-0.003, p=0.555; see Table S9). Focusing on mountain populations, it turns out that Alpine groups host the greatest Y chromosome among-population diversity. Interestingly, this does not hold only for German speakers and Ladins, who are the only groups subject to both geographic and linguistic isolation, but even for Italians, who show the apparently weakest signals of genetic drift.

**Table 2:** Within group diversity among population groups under study based on 5 Y chromosome STRs (DYS19, 390, 391, 392 and 393) and the hypervariable region of mtDNA (from 16033 to 16365).

	Y chromosome	mtDNA
German speakers	0.315 (0.184 - 0.380)	0.077 (0.057 - 0.091)
Italian speakers	0.053 (0.023 - 0.066)	0.008 (0.006 - 0.010)
Ladin speakers	0.077	0.035
North-Eastern Italy*	**-0.003**	**0.001**
Pyreneans	0.018 (0.002 - 0.031)	n.a.
South Tyroleans	0.004 (-0.006 - 0.011)	0.030 (0.014 - 0.046)

Values in brackets refer to minimum and maximum values obtained by jacknife procedure (see Table S9). Statistically insignificant values are in bold.

* This group is composed by three geographically close plain populations (Brescia, Treviso, Vicenza; see Table S4).

South Tyroleans provide an exception to the high and statistically significant Y-chromosome intra-group diversity of Alpine populations. A possible explanation for this finding comes from their particular social structure. In fact, since at least the early fourteenth century South Tyroleans have mostly complied with an inheritance and succession system known as *Geschlossener Hof* (“closed holding”), which entails an impartible transfer of the farm [52,53]. This system typically prescribes that only one son – generally the first born – takes over the economic unit consisting of the farmstead and the attached lands and succeeds into the position of a peasant house-father, while the other sons have the option to remain in the family farm as employees or to receive an economic compensation and relocate elsewhere [54,55,56,57]. Therefore, this practice may favor male dispersal, increasing the probability for sons other than the first born to marry far from the original community. Conversely, female mobility is less socially favored than in patrilocal groups. In the long term and under regimes of prevalent male mobility within the original groups, the *Geschlossener Hof* may lead to a pattern which is opposite to what would be expected for patrilocal groups. This is, in fact, the case of Tyrolean populations, who show only statistically significant intra-group variation for mtDNA polymorphisms (0.004, p=0.220 and 0.030 p=0 for Y chromosome and mtDNA respectively). Therefore, the *Geschlossener Hof* could have shaped intra-group variation of paternal lineages in the opposite way to “local ethnicity” [30]. If this was supported by further evidence, it would provide an example of divergent effects of socio-cultural factors on genetic diversity in populations who are closely related from a historical, linguistic and environmental point of view.

In conclusion, the comparison between Y chromosomal and mitochondrial patterns of variation suggests that not only geographic factors and linguistic diversity, but also socially induced sex biased gene flow should be taken into account when studying the genetic structure of Alpine populations. We believe this is an important avenue for any future research work which aims to shed light on the yet to be explored complexity of the genetic structure of European populations.

## Supporting Information

Figure S1 **Topology used for the simulations of evolutionary scenarios.**
(TIF)

Figure S2 **Multi-dimensional scaling plot of Fst genetic distances among Alpine populations based on mtDNA HVR-I sequences (stress value=0.153).** Acronyms are given in Table 1.
(TIF)

Figure S3 **Haplotype diversity of Alpine populations: (a) mitochondrial DNA values based on HVR-I region; (b) Y chromosome values based on 15 STRs (acronyms as in Table 1).**
(TIF)

Figure S4 **Analysis of molecular variance (AMOVA) within groups under study based on mtDNA sequences (hypervariable region 1) and 15 Y chromosome STRs.**
(TIF)

File S1 **17-loci Y-STR haplotypes and haplogroups of the populations under study.**
(XLS)

Table S1 **Literature data (Y chromosome 5 STRs) on populations settled in mountain ranges.**
(DOC)

Table S2 **Literature data on mtDNA (sequences of the hypervariable region 1) on Alpine populations.**
(DOC)

Table S3 **Analysis of molecular variance (AMOVA) among linguistic groups (Fst values below the diagonal, p-values above the diagonal).**
(DOC)

Table S4 **Literature data (Y chromosome 15 STRs) on European and Northern Italian open populations.**
(DOC)

Table S5 **Analysis of the molecular variance (AMOVA) within European and Northern Italy open populations groups based on 15 Y chromosome STRs (acronyms as in Table S4).**
(DOC)

Table S6 **Y chromosome (15 STRs) genetic diversity in 15 Alpine populations.**
(DOC)

Table S7 **Haplogroup frequency distribution in populations under study (acronyms as in Table 1).**
(DOC)

Table S8 **Mean census size and analysis of molecular variance (AMOVA) within Alpine linguistic groups under study based on 15 Y chromosome STRs (acronyms as in Table 1).**
(DOC)

Table S9 **Analysis of molecular variance (AMOVA) within groups under study based on 5 Y chromosome STRs, including results of jacknife procedure (acronyms as in Table 1, Table S1 and S4).**
(DOC)

## References

[1] Cavalli-Sforza LL, Menozzi P, Piazza A (1994) The History and Geography of Human Genes. New Jersey: Princeton University Press.

[2] Rosenberg NA, Pritchard JK, Weber JL, Cann HM, Kidd KK et al. (2002) Genetic structure of human populations. Science 298: 2381-2385. doi:10.1126/science.1078311. PubMed: 12493913. 12493913

[3] Busby GB, Brisighelli F, Sánchez-Diz P, Ramos-Luis E, Martinez-Cadenas C et al. (2012) The peopling of Europe and the cautionary tale of Y chromosome lineage R-M269. Proc Biol Sci 279: 884-892. doi:10.1098/rspb.2011.1044. PubMed: 21865258. 21865258 PMC3259916

[4] Rosser ZN, Zerjal T, Hurles ME, Adojaan M, Alavantic D et al. (2000) Y-chromosomal diversity in Europe is clinal and influenced primarily by geography, rather than by language. Am J Hum Genet 67: 1526-1543. doi:10.1086/316890. PubMed: 11078479. 11078479 PMC1287948

[5] Lao O, Lu TT, Nothnagel M, Junge O, Freitag-Wolf S et al. (2008) Correlation between genetic and geographic structure in Europe. Curr Biol 18: 1241-1248. doi:10.1016/j.cub.2008.07.049. PubMed: 18691889. 18691889

[6] Novembre J, Johnson T, Bryc K, Kutalik Z, Boyko AR et al. (2008) Genes mirror geography within Europe. Nature 456: 98-101. doi:10.1038/nature07331. PubMed: 18758442. 18758442 PMC2735096

[7] Seielstad MT, Minch E, Cavalli-Sforza LL (1998) Genetic evidence for a higher female migration rate in humans. Nat Genet 20: 278-280. doi:10.1038/3088. PubMed: 9806547. 9806547

[8] Fortunato L (2011) Reconstructing the history of residence strategies in Indo-European-speaking societies: neo-, uxori-, and virilocality. Hum Biol 83: 107-128. doi:10.3378/027.083.0107. PubMed: 21453007. 21453007

[9] Marks SJ, Levy H, Martinez-Cadenas C, Montinaro F, Capelli C (2012) Migration distance rather than migration rate explains genetic diversity in human patrilocal groups. Mol Ecol 21: 4958-4969. doi:10.1111/j.1365-294X.2012.05689.x. PubMed: 22765647. 22765647

[10] Bertranpetit J, Sala J, Calafell F, Underhill PA, Moral P et al. (1995) Human Mitochondrial DNA Variation and the Origin of Basques. Ann Hum Genet 59: 63-81. doi:10.1111/j.1469-1809.1995.tb01606.x. PubMed: 7762985. 7762985

[11] Thomas MG, Weale ME, Jones AL, Richards M, Smith A et al. (2002) Founding mothers of Jewish communities: geographically separated Jewish groups were independently founded by very few female ancestors. Am J Hum Genet 70: 1411-1420. doi:10.1086/340609. PubMed: 11992249. 11992249 PMC379128

[12] Fraumene C, Belle EMS, Castrì L, Sanna S, Mancosu G et al. (2006) High resolution analysis and phylogenetic network construction using complete mtDNA sequences in Sardinian genetic isolates. Mol Biol Evol 23: 2101–2111. doi:10.1093/molbev/msl084. PubMed: 16901986. 16901986

[13] Palo JU, Ulmanen I, Lukka M, Ellonen P, Sajantila A (2009) Genetic markers and population history: Finland revisited. Eur J Hum Genet 17: 1336-1346. doi:10.1038/ejhg.2009.53. PubMed: 19367325. 19367325 PMC2986642

[14] Novembre J, Ramachandran S (2011) Perspectives on human population structure at the cusp of the sequencing era. Annu Rev Genomics Hum Genet 12: 245-274. doi:10.1146/annurev-genom-090810-183123. PubMed: 21801023. 21801023

[15] Ostrer H, Skorecki K (2013) The population genetics of the Jewish people. Hum Genet 132: 119-127. doi:10.1007/s00439-012-1235-6. PubMed: 23052947. 23052947 PMC3543766

[16] Fedele F (1981) Il popolamento delle Alpi nel Paleolitico. Le Scienze 27: 22-39

[17] Straus LG (1991) Southwestern Europe and the Last Glacial Maximum. Curr Anthropol 32: 189-199. doi:10.1086/203940.

[18] Sauter MR (1979) Des chausseurs moustériens au Bas Empire. Schweizerische Zeitschrift für Geschichte 29: 125-143.

[19] Della Casa E (1999) Prehistoric alpine environment, society and economy. Bonn: Rudolf Habelt GmbH.

[20] Viazzo PP (1989) Upland communities. Environment, population and social structure in the Alps since the sixteenth century. Cambridge: Cambridge University Press.

[21] Vogel F (1992) Break-up of isolates. In: Roberts DF Fujiki N Torizuka K Isolation, migration and health. Cambridge: Cambridge University Press.

[22] Viazzo PP (2007) Transizioni alla modernità in area alpina. Dicotomie, paradossi, questioni aperte. Histoire des Alpes 12: 13-28.

[23] Toso F (2008) Le minoranze linguistiche in Italia. Bologna: Il Mulino.

[24] Steinicke E, Walder J, Löffler R, Beismann M (2011) Autochtonous linguistic minorities in the Italian Alps: new legislation – new identifications – new demographic processes. Journal Alpine Research: 99-92.

[25] Destro Bisol G, Anagnostou P, Batini C, Battaggia C, Bertoncini S et al. (2008) Italian isolates today: geographic and linguistic factors shaping human biodiversity. J Anthropol Sci 86: 179-188. PubMed: 19934475. 19934475

[26] Pichler I, Mueller JC, Stefanov SA, De Grandi A, Beu Volpato C et al. (2006) Genetic structure in contemporary South Tyrolean isolated populations revealed by analysis of Y-chromosome, mtDNA, and Alu polymorphisms. Hum Biol 78: 441-464. doi:10.1353/hub.2006.0057. PubMed: 17278620. 17278620

[27] Thomas MG, Barnes I, Weale ME, Jones AL, Forster P et al. (2008) New genetic evidence supports isolation and drift in the Ladin communities of the South Tyrolean alps but not an ancient origin in the Middle East. Eur J Hum Genet 16: 124-134. doi:10.1038/sj.ejhg.5201906. PubMed: 17712356. 17712356

[28] Boattini A, Griso C, Pettener D (2011) Are ethnic minorities synonymous for genetic isolates? Comparing Walser and Romance populations in the Upper Lys Valley (Western Alps). Anthropol Sci 89: 131-173. 10.4436/jass.89014 21757790

[29] Niederstätter H, Rampl G, Erhart D, Pitterl F, Oberacher H et al. (2012) Pasture names with Romance and Slavic roots facilitate dissection of Y chromosome variation in an exclusively German-speaking alpine region. PLOS ONE 7: e41885. doi:10.1371/journal.pone.0041885. PubMed: 22848647. 22848647 PMC3407130

[30] Capocasa M, Battaggia C, Anagnostou P, Montinaro F, Boschi I et al. (2013) Detecting genetic isolation in human populations: a study of European language minorities. PLOS ONE 8: e56371. doi:10.1371/journal.pone.0056371. PubMed: 23418562. 23418562 PMC3572090

[31] Belardi W (1994) Profilo storico-politico della lingua e della letteratura ladina. Roma: Il calamo.

[32] Congiu A, Anagnostou P, Milia N, Capocasa M, Montinaro F et al. (2012) Online databases for mtDNA and Y chromosome polymorphisms in human populations. J Anthropol Sci 90: 197-212. PubMed: 23274751. 10.4436/jass.90020 23274751

[33] Onofri V, Alessandrini F, Turchi C, Pesaresi M, Buscemi L et al. (2006) Development of multiplex PCRs for evolutionary and forensic applications of 37 human Y chromosome SNPs. Forensic Sci Int 157: 23-35. doi:10.1016/j.forsciint.2005.03.014. PubMed: 15896936. 15896936

[34] Ferri G, Alù M (2012) Development of six-y-SNPs assay for forensic analysis in European population. DNA in Forensics 2012, 5th International EMPOP Meeting-8th International Forensic Y-User Workshop, Innsbruck

[35] Alvarez-Iglesias V, Mosquera-Miguel A, Cerezo M, Quintáns B, Zarrabeitia MT et al. (2009) New population and phylogenetic features of the internal variation within mitochondrial DNA macro-haplogroup R0. PLOS ONE 4: e5112. doi:10.1371/journal.pone.0005112. PubMed: 19340307. 19340307 PMC2660437

[36] Bell JR (2008) A simple way to treat PCR products prior to sequencing using ExoSAP-IT. BioTechniques 44: 834. doi:10.2144/000112890. PubMed: 18476841. 18476841

[37] Reynolds J, Weir BS, Cockerham CC (1983) Estimation for the coancestry coefficient: basis for a short-term genetic distance. Genetics 105: 767-779. PubMed: 17246175. 17246175 10.1093/genetics/105.3.767 PMC1202185

[38] Excoffier L, Lischer HEL (2010) Arlequin suite ver 3.5: a new series of programs to perform population genetics analyses under Linux and Windows. Mol Ecol Resour 10: 564-567. doi:10.1111/j.1755-0998.2010.02847.x. PubMed: 21565059. 21565059

[39] Tofanelli S, Taglioli L, Merlitti D, Paoli G (2011) Tools which simulate the evolution of uni-parentally transmitted elements of the human genome. J Anthropol Sci 89: 201-219. PubMed: 21911915. 21911915 10.4436/jass.89017

[40] Coia V, Boschi I, Trombetta F, Cavulli F, Montinaro F et al. (2012) Evidence of high genetic variation among linguistically diverse populations on a micro-geographic scale: a case study of the Italian Alps. J Hum Genet 57: 254-260. doi:10.1038/jhg.2012.14. PubMed: 22418692. 22418692

[41] Bramanti B, Thomas MG, Haak W, Unterlaender M, Jores P et al. (2009) Genetic discontinuity between local hunter-gatherers and central Europe's first farmers. Science 326: 137-140. doi:10.1126/science.1176869. PubMed: 19729620. 19729620

[42] Rasteiro R, Chikhi L (2013) Female and male perspectives on the neolithic transition in Europe: clues from ancient and modern genetic data. PLOS ONE 8: e60944. doi:10.1371/journal.pone.0060944. PubMed: 23613761. 23613761 PMC3629215

[43] Ballantyne KN, Goedbloed M, Fang R, Schaap O, Lao O et al. (2010) Mutability of Y-chromosomal microsatellites: rates, characteristics, molecular bases, and forensic implications. Am J Hum Genet 87: 341-353. doi:10.1016/j.ajhg.2010.08.006. PubMed: 20817138. 20817138 PMC2933352

[44] Moz NA (2011) Luserna. Terra di uomini liberi. Rovereto: Osiride.

[45] Cruciani F, Trombetta B, Antonelli C, Pascone R, Valesini G et al. (2011) Strong intra- and inter-continental differentiation revealed by Y chromosome SNPs M269, U106 and U152. Forensic Sci Int Genet 5: e49-e52. doi:10.1016/j.fsigen.2010.07.006. PubMed: 20732840. 20732840

[46] Pescosta W (2010) Storia dei ladini delle Dolomiti. Istitut Ladin Micura´De Rü.

[47] Lanzinger M, Marzatico F, Pedrotti A (2000) Storia del Trentino. Vol. I. La preistoria e la protostoria. Bologna: Il Mulino.

[48] Viazzo PP (2009) Le comunità walser del Monte Rosa tra XVIII e XIX secolo: demografia, economia e migrazioni. In: Viazzo PP Cerri R Da montagna a montagna. Mobilità e migrazioni interne nelle Alpi italiane (secoli XVII-XIX). Alagna-Magenta. Edizioni Zeisciu Centro Studi pp. 65-83.

[49] Rapelli G (2004) XIII comuni veronesi. La formazione dell'isola linguistica. Pezzi C, editor Isole di cultura. Saggi sulle minoranze storiche germaniche in Italia. Luserna: Comitato Unitario delle Isole Linguistiche Storiche Germaniche in Italia-Centro Documentazione Luserna. pp. 243-248

[50] Roewer L, Croucher PJ, Willuweit S, Lu TT, Kayser M et al. (2005) Signature of recent historical events in the European Y-chromosomal STR haplotype distribution. Hum Genet 116: 279-291. doi:10.1007/s00439-004-1201-z. PubMed: 15660227. 15660227

[51] López-Parra AM, Gusmão L, Tavares L, Baeza C, Amorim A et al. (2009) In search of the Pre- and Post-Neolithic genetic substrates in Iberia: evidence from Y-chromosome in Pyrenean populations. Ann Hum Genet 73: 42-53. doi:10.1111/j.1469-1809.2008.00478.x. PubMed: 18803634. 18803634

[52] Winkler W (1980) Anerbenrecht. In: Lexikon des Mittelalters (Vol. 1). München: Artemis Verlag pp. 616-617.

[53] Schennach MP (2003) Geschichte des bäuerlichen Besitz- und Erbrechts in Tirol. In: Amt der Tiroler Landesregierung, Abt; Landesarchiv Tiroler, Hofgeschichten der (2002) und 2003 verliehenen Erbhöfe (Tiroler Erbhöfe No. 21). Innsbruck

[54] Cole JW, Wolf ER (1974) The hidden frontier. Ecology and ethnicity in an Alpine valley. New York: Academic Press.

[55] Pavanello M (2007). . Breve introduzione allo studio antropologico della parentela. Roma Edizioni Nuova Cultura.

[56] Ehmer J (2009) House and the stem family in Austria. In: Fauve-Chamoux A Ochiai E The stem family in Eurasian perspective. Bern: Peter Lang pp. 103-131.

[57] Mori E, Hintner W (2009) Il maso chiuso del Sudtirolo: la sua storia e la normativa vigente. Bolzano: Provincia autonoma.

