# Supplementary figures and images for: Demographic Histories, Isolation and Social Factors as Determinants of the Genetic Structure of Alpine Linguistic Groups
Source: PLoS One. 2013 Dec 2;8(12):e81704. doi: 10.1371/journal.pone.0081704 (PMC3847036; doi:10.1371/journal.pone.0081704)

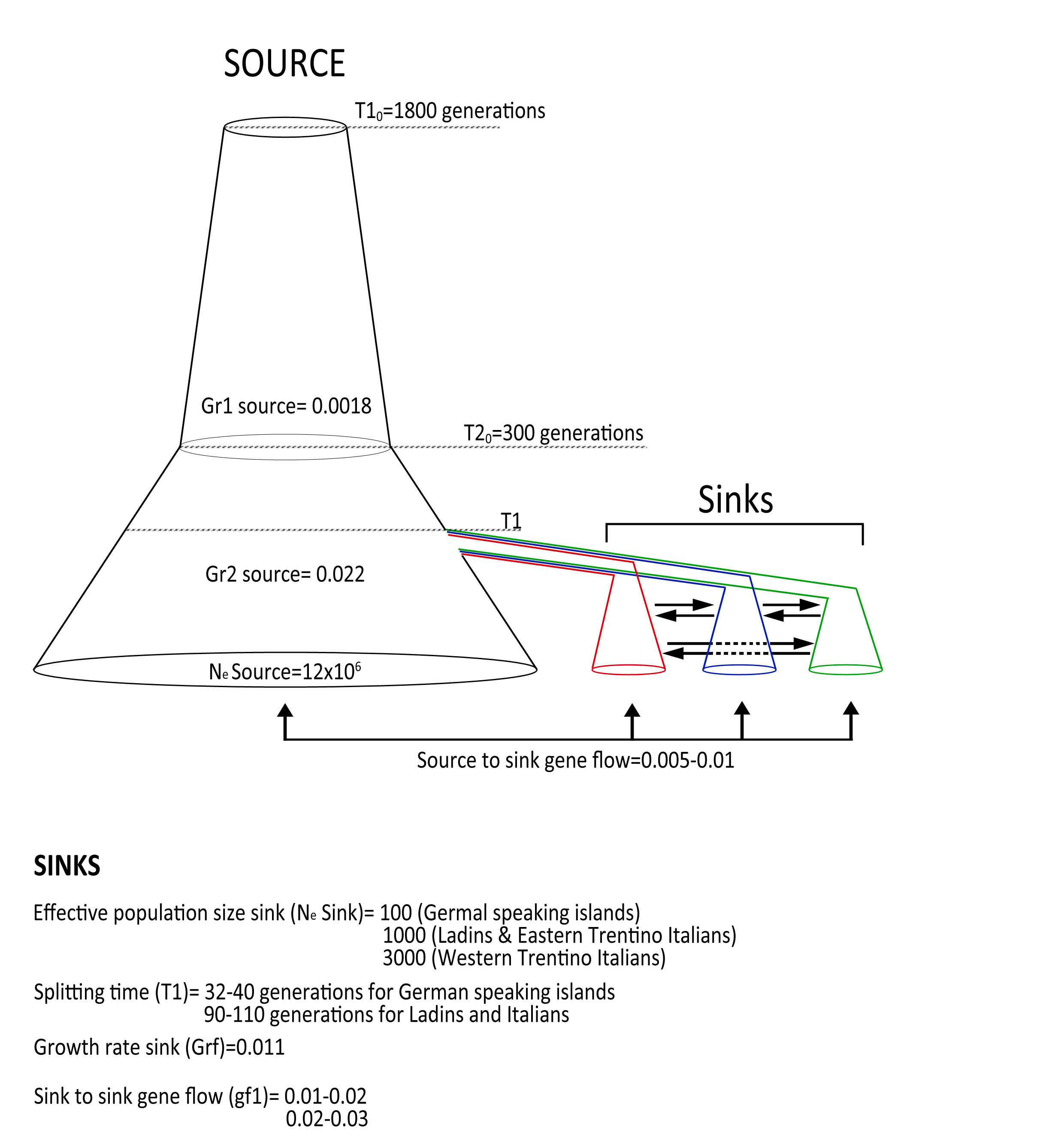

Supplement: Figure S1 — Topology used for the simulations of evolutionary scenarios. (TIF) [file pone.0081704.s001.tif]

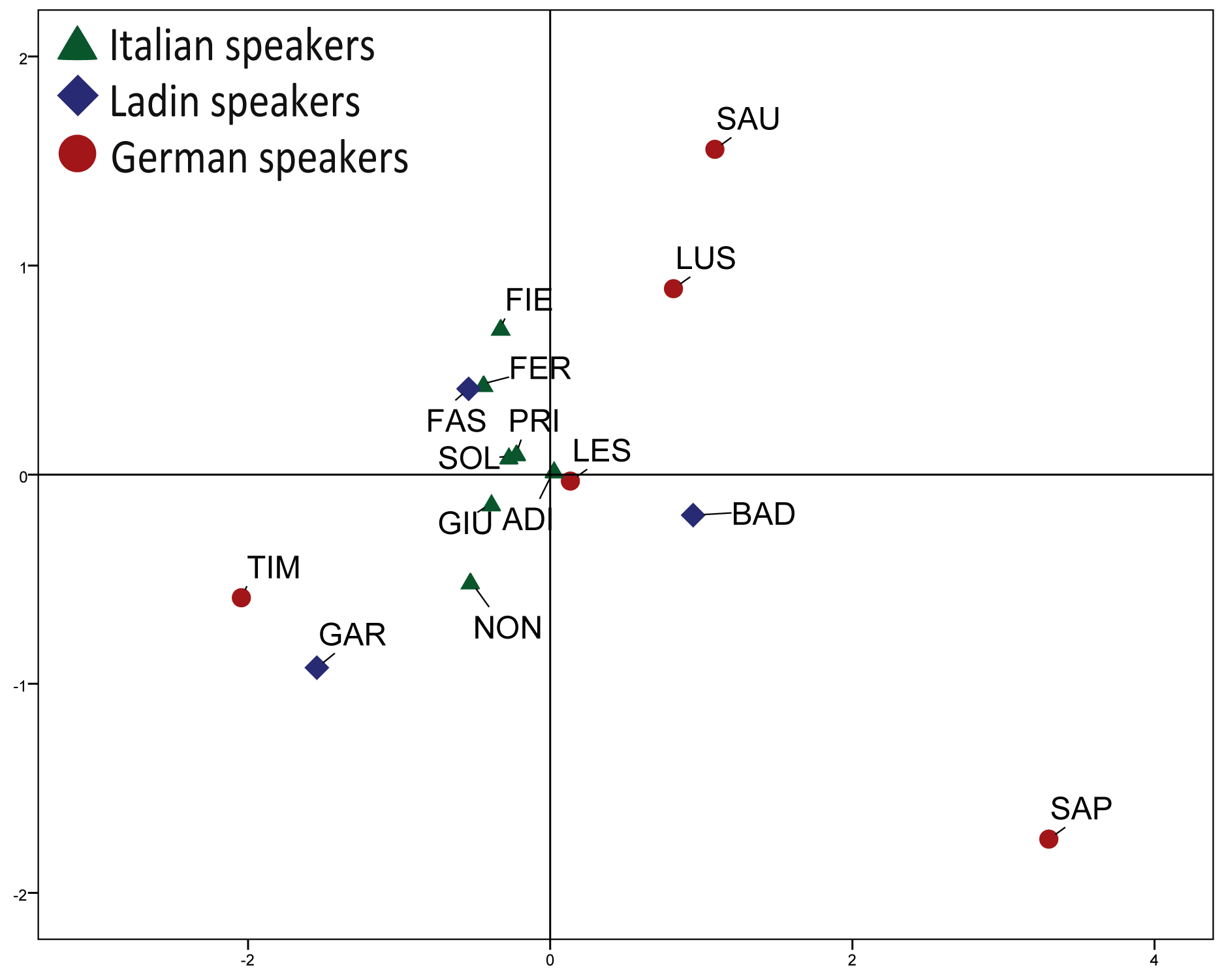

Supplement: Figure S2 — Multi-dimensional scaling plot of Fst genetic distances among Alpine populations based on mtDNA HVR-I sequences (stress value=0.153). Acronyms are given in Table 1. (TIF) [file pone.0081704.s002.tif]

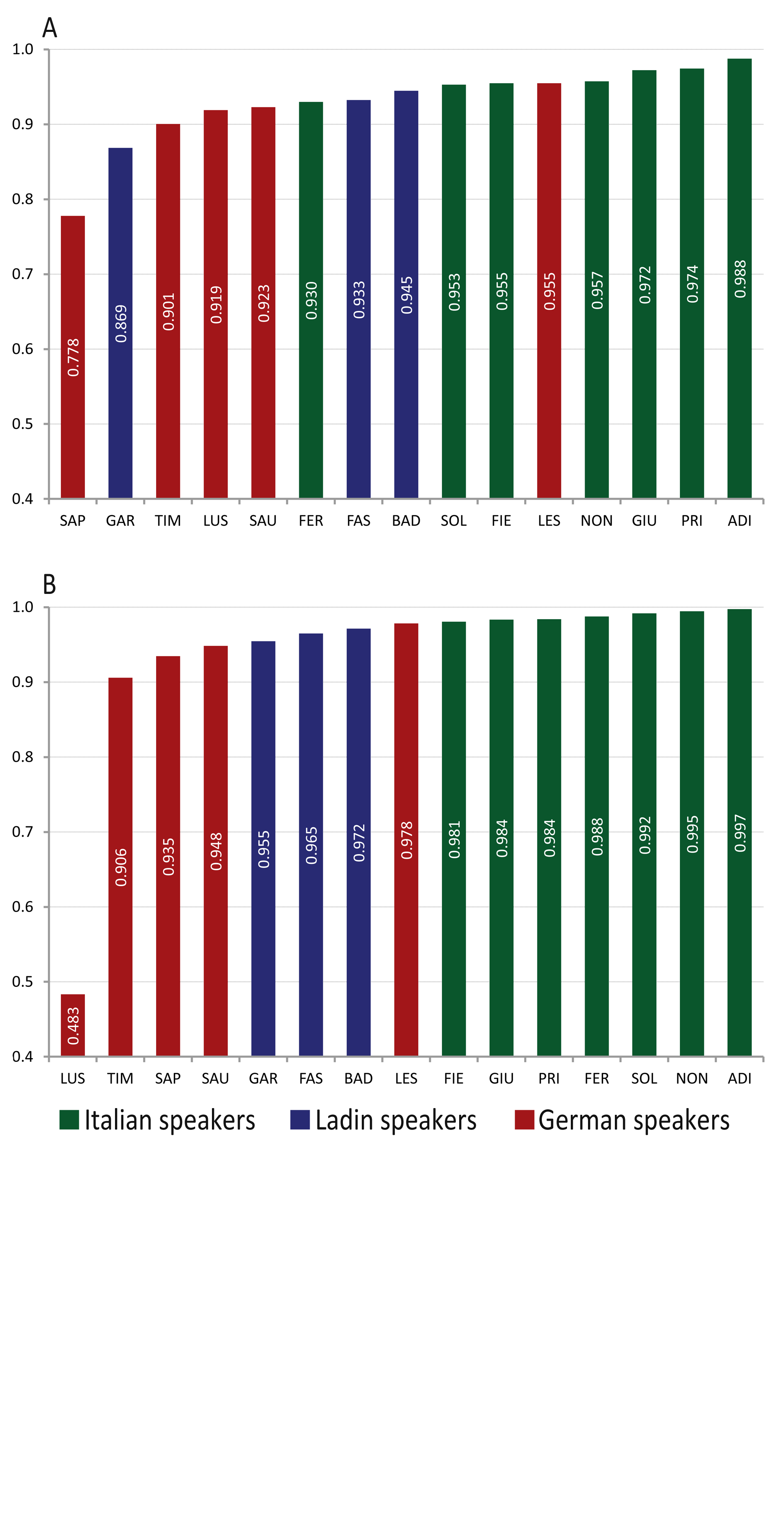

Supplement: Figure S3 — Haplotype diversity of Alpine populations: (a) mitochondrial DNA values based on HVR-I region; (b) Y chromosome values based on 15 STRs (acronyms as in Table 1). (TIF) [file pone.0081704.s003.tif]

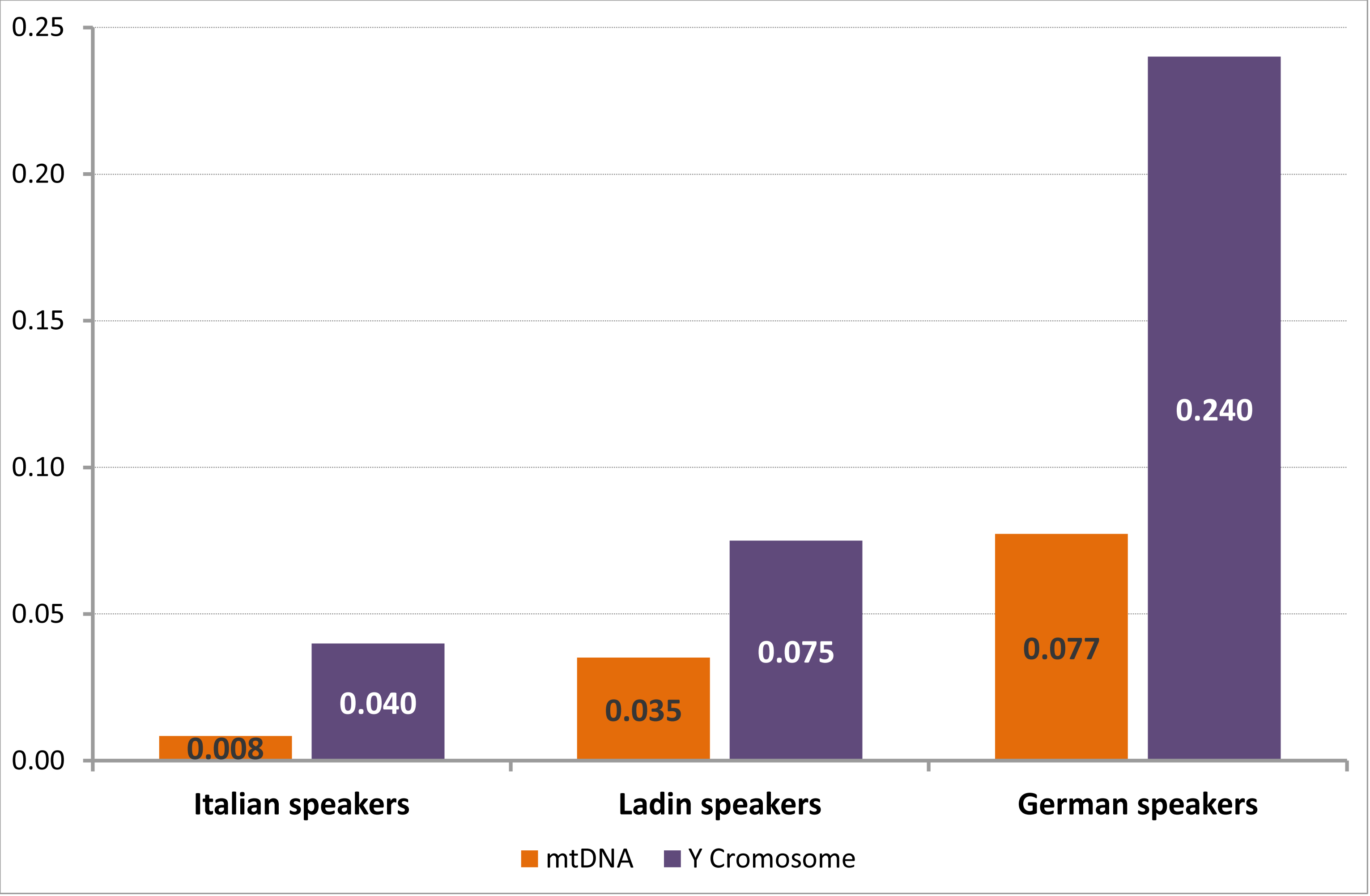

Supplement: Figure S4 — Analysis of molecular variance (AMOVA) within groups under study based on mtDNA sequences (hypervariable region 1) and 15 Y chromosome STRs. (TIF) [file pone.0081704.s004.tif]
