# Supplementary material for: Demographic Histories, Isolation and Social Factors as Determinants of the Genetic Structure of Alpine Linguistic Groups
Source: PLoS One. 2013 Dec 2;8(12):e81704. doi: 10.1371/journal.pone.0081704 (PMC3847036; doi:10.1371/journal.pone.0081704)
Supplement: Table S1 — Literature data (Y chromosome 5 STRs) on populations settled in mountain ranges. (DOC) [file pone.0081704.s006.doc]

**Supplementary Table S1.** Literature data (Y chromosome 5 STRs) on populations settled in mountain ranges.

| **Population** | **Mountain range** | **Abbreviation** | **Sample size** | **Reference** |
| --- | --- | --- | --- | --- |
| Alto Urgel | Pyrenean | URG | 34 | Lopez-Parra et al 2009 |
| Cerdana | Pyrenean | CER | 37 | Lopez-Parra et al 2009 |
| Cinco Villas | Pyrenean | CIN | 42 | Lopez-Parra et al 2009 |
| Isarco valley | Alps | ISA | 34 | Pichler et al 2006 |
| Jacetania | Pyrenean | JAC | 31 | Lopez-Parra et al 2009 |
| Lower Venosta valley | Alps | VEL | 52 | Thomas et al 2008 |
| Pusteria valley | Alps | PUS | 35 | Pichler et al 2006 |
| Upper Venosta valley | Alps | VEU | 50 | Thomas et al 2008 |
| Valle de Aràn | Pyrenean | ARA | 25 | Lopez-Parra et al 2009 |

**References**

Lopez-Parra AM, Gusmao L, Tavares L, Baeza C, Amorim A, et al. (2009) In search of the Pre- and Post-Neolithic genetic substrates in Iberia: evidence from Y-chromosome in Pyrenean populations. Ann Hum Genet 73: 42-53.

Pichler I, Mueller JC, Stefanov SA, De Grandi A, Beu Volpato C, et al. (2006) Genetic structure in contemporary South Tyrolean isolated populations revealed by analysis of Y-chromosome, mtDNA, and Alu polymorphisms. Hum Biol 78: 441-464.

Thomas MG, Barnes I, Weale ME, Jones AL, Forster P, et al. (2008) New genetic evidence supports isolation and drift in the Ladin communities of the South Tyrolean alps but not an ancient origin in the Middle East. Eur J Hum Genet 16: 124-134.
