# Supplementary material for: Demographic Histories, Isolation and Social Factors as Determinants of the Genetic Structure of Alpine Linguistic Groups
Source: PLoS One. 2013 Dec 2;8(12):e81704. doi: 10.1371/journal.pone.0081704 (PMC3847036; doi:10.1371/journal.pone.0081704)
Supplement: Table S2 — Literature data on mtDNA (sequences of the hypervariable region 1) on Alpine populations. (DOC) [file pone.0081704.s007.doc]

**Supplementary Table S2.** Literature data (mtDNA HVR-1) on Alpine populations.

| **Population (region)** | **Abbreviation** | **Sample size** | **Reference** |
| --- | --- | --- | --- |
| Adige (Trentino) | ADI | 56 | Coia et al 2012 |
| Badia (South Tyrol) | BAD | 56 | Thomas et al 2008 |
| Fassa (Trentino) | FAS | 47 | Coia et al 2012 |
| Fersina (Trentino) | FER | 25 | Coia et al 2012 |
| Fiemme (Trentino) | FIE | 41 | Coia et al 2012 |
| Gardena (South Tyrol) | GAR | 46 | Thomas et al 2008 |
| Giudicarie (Trentino) | GIU | 52 | Coia et al 2012 |
| Isarco valley (South Tyrol) | ISA | 34 | Pichler et al 2006 |
| Lessinia (Veneto) | LES | 41 | Capocasa et al 2013 |
| Lower Venosta valley (South Tyrol) | VEL | 52 | Thomas et al 2008 |
| Luserna (Trentino) | LUS | 21 | Coia et al 2012 |
| Non (Trentino) | NON | 48 | Coia et al 2012 |
| Primiero (Trentino) | PRI | 40 | Coia et al 2012 |
| Pusteria valley (South Tyrol) | PUS | 37 | Pichler et al 2006 |
| Sappada (Veneto) | SAP | 59 | Capocasa et al 2013 |
| Sauris (Friuli) | SAU | 48 | Capocasa et al 2013 |
| Sole (Trentino) | SOL | 63 | Coia et al 2012 |
| Timau (Friuli) | TIM | 46 | Capocasa et al 2013 |
| Upper Venosta valley (South Tyrol) | VEU | 50 | Thomas et al 2008 |

**References**

Capocasa M, Battaggia C, Anagnostou P, Montinaro F, Boschi I, et al. (2013) Detecting genetic isolation in human populations: a study of European language minorities. PLoS One 8: e56371.

Coia V, Boschi I, Trombetta F, Cavulli F, Montinaro F, et al. (2012) Evidence of high genetic variation among linguistically diverse populations on a micro-geographic scale: a case study of the Italian Alps. J Hum Genet 57: 254-260.

Pichler I, Mueller JC, Stefanov SA, De Grandi A, Beu Volpato C, et al. (2006) Genetic structure in contemporary South Tyrolean isolated populations revealed by analysis of Y-chromosome, mtDNA, and Alu polymorphisms. Hum Biol 78: 441-464.

Thomas MG, Barnes I, Weale ME, Jones AL, Forster P, et al. (2008) New genetic evidence supports isolation and drift in the Ladin communities of the South Tyrolean alps but not an ancient origin in the Middle East. Eur J Hum Genet 16: 124-134.
