# Supplementary material for: Demographic Histories, Isolation and Social Factors as Determinants of the Genetic Structure of Alpine Linguistic Groups
Source: PLoS One. 2013 Dec 2;8(12):e81704. doi: 10.1371/journal.pone.0081704 (PMC3847036; doi:10.1371/journal.pone.0081704)
Supplement: Table S3 — Analysis of molecular variance (AMOVA) among linguistic groups (Fst values below the diagonal, p-values above the diagonal). (DOC) [file pone.0081704.s008.doc]

**Supplementary Table S3.** Analysis of molecular variance (AMOVA) among linguistic groups (Fst values below the diagonal, p-values above the diagonal).

| **15 Y chromosomal STRs** | |  |  |
| --- | --- | --- | --- |
|  | Italian speakers | German speakers | Ladin speakers |
| Italian speakers | - | 0.021 | 0.094 |
| German speakers | 0.020 | - | 0.129 |
| Ladin speakers | 0.007 | 0.012 | - |
|  |  |  |  |
| **Mitochondrial DNA, hypervariable region 1** | | |  |
|  | Italian speakers | German speakers | Ladin speakers |
| Italian speakers | - | 0.065 | 0.583 |
| German speakers | 0.600 | - | 0.847 |
| Ladin speakers | -0.080 | -0.780 | - |
