# Supplementary material for: Demographic Histories, Isolation and Social Factors as Determinants of the Genetic Structure of Alpine Linguistic Groups
Source: PLoS One. 2013 Dec 2;8(12):e81704. doi: 10.1371/journal.pone.0081704 (PMC3847036; doi:10.1371/journal.pone.0081704)
Supplement: Table S4 — Literature data (Y chromosome 15 STRs) on European and Northern Italian open populations. (DOC) [file pone.0081704.s009.doc]

**Supplementary Table S4.** Literature data (Y chromosome 15 STRs) on European and Northern Italian open populations.

| **Population** | **Abbreviation** | **Sample size** | **Reference** |
| --- | --- | --- | --- |
| Austria | AUS | 134 | Berger et al 2005 |
| Basques (Spain) | BAS | 139 | Valverde et al 2011 |
| Brescia (Italy) | BRE | 35 | Boattini et al 2013 |
| Como (Italy) | COM | 41 | Boattini et al 2013 |
| Croatia | CRO | 165 | Ljubkovic et al 2008 |
| Cuneo (Italy) | CUN | 30 | Boattini et al 2013 |
| Finns | FIN | 907 | Palo et al 2009 |
| La Spezia (Italy) | SPE | 24 | Boattini et al 2013 |
| Poland | POL | 255 | Soltyszewski et al 2007 |
| Portugal (central) | POR | 99 | Sánchez-Diz et al 2008 |
| Sardinians (Italy) | SAR | 78 | Boattini et al 2013 |
| Serbia | SER | 185 | Veselinovic et al 2008 |
| Spain | SPA | 240 | Sánchez et al 2007 |
| Treviso (Italy) | TRE | 28 | Boattini et al 2013 |
| Vicenza (Italy) | VIC | 33 | Boattini et al 2013 |

**References**

Berger B, Lindinger A, Niederstatter H, Grubwieser P, Parson W (2005) Y-STR typing of an Austrian population sample using a 17-loci multiplex PCR assay. Int J Legal Med 119: 241-246.

Boattini A, Martinez-Cruz B, Sarno S, Harmant C, Useli A, et al. (2013) Uniparental markers in Italy reveal a sex-biased genetic structure and different historical strata. PLoS One 8: e65441.

Ljubković J, Stipisić A, Sutlović D, Definis-Gojanović M, Bucan K, et al. (2008) Y-chromosomal short tandem repeat haplotypes in southern Croatian male population defined by 17 loci. Croat Med J 49: 201-206.

[Palo JU](http://www.ncbi.nlm.nih.gov/pubmed?term=Palo JU%5BAuthor%5D&cauthor=true&cauthor_uid=19367325), [Ulmanen I](http://www.ncbi.nlm.nih.gov/pubmed?term=Ulmanen I%5BAuthor%5D&cauthor=true&cauthor_uid=19367325), [Lukka M](http://www.ncbi.nlm.nih.gov/pubmed?term=Lukka M%5BAuthor%5D&cauthor=true&cauthor_uid=19367325), [Ellonen P](http://www.ncbi.nlm.nih.gov/pubmed?term=Ellonen P%5BAuthor%5D&cauthor=true&cauthor_uid=19367325), [Sajantila A](http://www.ncbi.nlm.nih.gov/pubmed?term=Sajantila A%5BAuthor%5D&cauthor=true&cauthor_uid=19367325) (2009) Genetic markers and population history: Finland revisited. [Eur J Hum Genet.](http://www.ncbi.nlm.nih.gov/pubmed/19367325) 17:1336-1346.

Sánchez C, Barrot C, Xifró A, Ortega M, de Aranda IG, et al. (2007) Haplotype frequencies of 16 Y-chromosome STR loci in the Barcelona metropolitan area population using Y-Filer kit. Forensic Sci Int 172: 211-217.

Sánchez-Diz P, Alves C, Carvalho E, Carvalho M, Espinheira R, et al. (2008) Population and segregation data on 17 Y-STRs: results of a GEP-ISFG collaborative study. Int J Legal Med 122: 529-533.

Soltyszewski I, Pepinski W, Spolnicka M, Kartasinska E, Konarzewska M, et al. (2007) Y-chromosomal haplotypes for the AmpFISTR Yfiler PCR Amplification Kit in a population sample from Central Poland. Forensic Sci Int 168: 61-67.

Valverde L, Rosique M, Köhnemann S, Cardoso S, García-Gasca A, et al. (2011) Y-STR variation in the Basque Diaspora in the Western US: evolutionary and forensic perspectives. Int J Legal Med 126: 293-298.

Veselinovic IS, Zgonjanin DM, Maletin MP, Stojkovic O, Djurendic-Brenesel M, et al. (2008) Allele frequencies and population data for 17 Y-chromosome STR loci in a Serbian population sample from Vojvodina province. Forensic Sci Int 176: e23-e28.
