# Supplementary material for: Demographic Histories, Isolation and Social Factors as Determinants of the Genetic Structure of Alpine Linguistic Groups
Source: PLoS One. 2013 Dec 2;8(12):e81704. doi: 10.1371/journal.pone.0081704 (PMC3847036; doi:10.1371/journal.pone.0081704)
Supplement: Table S5 — Analysis of the molecular variance (AMOVA) within European and Northern Italy open populations groups based on 15 Y chromosome STRs (acronyms as in Table S4). (DOC) [file pone.0081704.s010.doc]

**Supplementary Table S5.** Analysis of the molecular variance (AMOVA) within European and Northern Italy open populations groups based on 15 Y chromosome STRs (acronyms as in Supplementary Table S4).

| **Group** | **Populations** | **Within group** | **p value** |
| --- | --- | --- | --- |
| Europe | AUS-CRO-POL-POR-SER-SPA | 0.074 | 0.000 |
|  | Excluding AUS | 0.084 | 0.000 |
|  | Excluding CRO | 0.070 | 0.000 |
|  | Excluding POL | 0.061 | 0.000 |
|  | Excluding POR | 0.075 | 0.000 |
|  | Excluding SER | 0.086 | 0.000 |
|  | Excluding SPA | 0.065 | 0.000 |
| Northern Italy | BRE-COM-CUN-SPE-TRE-VIC | 0.006 | 0.101 |
|  | Excluding BRE | 0.007 | 0.134 |
|  | Excluding COM | 0.011 | 0.052 |
|  | Excluding CUN | 0.011 | 0.041 |
|  | Excluding SPE | 0.005 | 0.169 |
|  | Excluding TRE | 0.009 | 0.066 |
|  | Excluding VIC | -0.005 | 0.806 |
