# Supplementary material for: Demographic Histories, Isolation and Social Factors as Determinants of the Genetic Structure of Alpine Linguistic Groups
Source: PLoS One. 2013 Dec 2;8(12):e81704. doi: 10.1371/journal.pone.0081704 (PMC3847036; doi:10.1371/journal.pone.0081704)
Supplement: Table S6 — Y chromosome (15 STRs) genetic diversity in 15 Alpine populations. (DOC) [file pone.0081704.s011.doc]

**Supplementary Table S6.** Y chromosome (15 STRs) genetic diversity in 15 Alpine populations.

| **Population (region)** | **Abbreviation** | **Sample size** | **N° of haplotypes** | **HD** | **HD s.d.** | **Average Fst** |
| --- | --- | --- | --- | --- | --- | --- |
| Adige (Trentino) | ADI | 56 | 53 | 0.997 | 0.004 | 0.071 |
| Badia (South Tyrol) | BAD | 44 | 28 | 0.972 | 0.012 | 0.106 |
| Fassa (Trentino) | FAS | 47 | 29 | 0.965 | 0.013 | 0.088 |
| Fersina (Trentino) | FER | 26 | 22 | 0.988 | 0.014 | 0.088 |
| Fiemme (Trentino) | FIE | 41 | 30 | 0.981 | 0.011 | 0.083 |
| Gardena (South Tyrol) | GAR | 51 | 30 | 0.955 | 0.016 | 0.120 |
| Giudicarie (Trentino) | GIU | 51 | 41 | 0.984 | 0.010 | 0.088 |
| Lessinia (Veneto) | LES | 24 | 19 | 0.978 | 0.019 | 0.097 |
| Luserna (Trentino) | LUS | 25 | 6 | 0.483 | 0.119 | 0.328 |
| Non (Trentino) | NON | 48 | 42 | 0.995 | 0.005 | 0.078 |
| Primiero (Trentino) | PRI | 41 | 32 | 0.984 | 0.010 | 0.114 |
| Sappada (Veneto) | SAP | 38 | 22 | 0.935 | 0.029 | 0.181 |
| Sauris (Friuli) | SAU | 29 | 19 | 0.948 | 0.028 | 0.092 |
| Sole (Trentino) | SOL | 65 | 52 | 0.992 | 0.005 | 0.069 |
| Timau (Friuli) | TIM | 24 | 11 | 0.906 | 0.032 | 0.214 |

Abbreviations: HD. haplotype diversity; s.d. standard deviation.
