# Supplementary material for: Demographic Histories, Isolation and Social Factors as Determinants of the Genetic Structure of Alpine Linguistic Groups
Source: PLoS One. 2013 Dec 2;8(12):e81704. doi: 10.1371/journal.pone.0081704 (PMC3847036; doi:10.1371/journal.pone.0081704)
Supplement: Table S7 — Haplogroup frequency distribution in populations under study (acronyms as in Table 1). (DOC) [file pone.0081704.s012.doc]

**Table S7.** Haplogroup frequency distribution in populations under study (acronyms as in Table 1).

| **Haplogroup** | ADI | BAD | FAS | FER | FIE | GAR | GIU | LES | LUS | NON | PRI | SAP | SAU | SOL | TIM |
| --- | --- | --- | --- | --- | --- | --- | --- | --- | --- | --- | --- | --- | --- | --- | --- |
| E1b-M35 | 0 | 0 | 0 | 0 | 0 | 0.02 | 0 | 0 | 0 | 0 | 0 | 0 | 0 | 0 | 0 |
| E1b-M521 | 0 | 0 | 0 | 0 | 0 | 0 | 0.02 | 0 | 0 | 0 | 0 | 0 | 0 | 0 | 0 |
| E1b-M78* | 0 | 0 | 0 | 0.077 | 0 | 0 | 0 | 0 | 0 | 0.042 | 0 | 0 | 0 | 0 | 0 |
| E1b-V12 | 0 | 0 | 0 | 0 | 0 | 0 | 0 | 0 | 0 | 0.042 | 0 | 0 | 0 | 0.015 | 0 |
| E1b-V13* | 0.107 | 0 | 0.021 | 0.077 | 0.026 | 0.059 | 0.039 | 0 | 0 | 0.125 | 0.024 | 0.632 | 0 | 0.092 | 0 |
| E1b-V22* | 0 | 0 | 0 | 0.038 | 0 | 0 | 0 | 0 | 0 | 0 | 0.024 | 0 | 0.138 | 0 | 0 |
| G-M201 | 0.054 | 0.091 | 0.043 | 0.115 | 0.051 | 0.039 | 0.059 | 0.103 | 0 | 0.104 | 0.488 | 0.105 | 0.034 | 0.108 | 0 |
| I-M170* | 0 | 0.023 | 0 | 0 | 0 | 0 | 0 | 0 | 0 | 0 | 0 | 0 | 0 | 0 | 0 |
| I1-M253 | 0.036 | 0.068 | 0 | 0 | 0.077 | 0.078 | 0.02 | 0.207 | 0 | 0.021 | 0.024 | 0 | 0.069 | 0.062 | 0.13 |
| I2-M223 | 0.018 | 0 | 0 | 0 | 0 | 0 | 0 | 0 | 0 | 0 | 0 | 0 | 0.241 | 0 | 0 |
| I2-P37.2 | 0.036 | 0 | 0 | 0 | 0 | 0.02 | 0.02 | 0 | 0 | 0 | 0.024 | 0 | 0 | 0 | 0 |
| J1-M267 | 0 | 0 | 0.149 | 0 | 0.026 | 0 | 0 | 0 | 0 | 0 | 0.024 | 0 | 0 | 0 | 0 |
| J2a-M172* | 0 | 0 | 0 | 0 | 0 | 0.02 | 0 | 0 | 0 | 0 | 0 | 0 | 0 | 0 | 0 |
| J2a-M410* | 0.018 | 0.045 | 0.043 | 0 | 0.051 | 0.157 | 0.059 | 0.241 | 0 | 0 | 0 | 0.079 | 0.103 | 0.123 | 0 |
| J2a-L27 (M319+ M67 + m92) | 0 | 0 | 0.043 | 0.038 | 0 | 0.02 | 0.078 | 0 | 0 | 0.021 | 0 | 0 | 0 | 0.015 | 0 |
| J2b-M102* | 0.018 | 0 | 0.085 | 0 | 0.051 | 0.02 | 0.118 | 0 | 0 | 0.021 | 0.024 | 0 | 0 | 0.015 | 0.304 |
| K-M9 | 0.036 | 0.091 | 0.234 | 0 | 0.077 | 0 | 0 | 0.138 | 0.08 | 0 | 0 | 0 | 0 | 0.062 | 0 |
| R1a-M17 | 0.071 | 0 | 0.021 | 0.038 | 0 | 0.02 | 0 | 0 | 0 | 0.083 | 0.073 | 0 | 0.034 | 0 | 0.565 |
| R1b-M269* | 0.054 | 0 | 0.021 | 0.038 | 0.154 | 0 | 0.137 | 0 | 0.84 | 0.104 | 0.098 | 0 | 0 | 0.031 | 0 |
| R1b-S116* | 0.071 | 0 | 0 | 0 | 0.026 | 0 | 0 | 0.172 | 0 | 0.063 | 0.024 | 0.053 | 0 | 0 | 0 |
| R1b-S127* | 0.018 | 0.023 | 0 | 0 | 0 | 0 | 0 | 0.034 | 0.04 | 0.021 | 0 | 0 | 0 | 0 | 0 |
| R1b-S139* | 0.071 | 0.477 | 0.255 | 0.038 | 0.205 | 0.078 | 0.039 | 0.069 | 0 | 0.063 | 0.073 | 0 | 0.345 | 0.031 | 0 |
| R1b-S145* | 0.018 | 0.045 | 0.021 | 0 | 0 | 0.235 | 0 | 0 | 0 | 0 | 0 | 0 | 0 | 0 | 0 |
| R1b-S167* | 0.018 | 0.045 | 0.021 | 0 | 0.051 | 0.176 | 0 | 0 | 0 | 0 | 0 | 0.132 | 0 | 0.031 | 0 |
| R1b-S21* | 0.036 | 0.068 | 0.021 | 0.077 | 0.179 | 0.039 | 0 | 0 | 0 | 0.104 | 0 | 0 | 0.034 | 0.108 | 0 |
| R1b-S28* | 0.286 | 0.023 | 0.021 | 0.385 | 0.026 | 0.02 | 0.412 | 0.034 | 0 | 0.188 | 0.098 | 0 | 0 | 0.308 | 0 |
| R1b-SRY2627 | 0.036 | 0 | 0 | 0.077 | 0 | 0 | 0 | 0 | 0.04 | 0 | 0 | 0 | 0 | 0 | 0 |
