# Supplementary material for: Demographic Histories, Isolation and Social Factors as Determinants of the Genetic Structure of Alpine Linguistic Groups
Source: PLoS One. 2013 Dec 2;8(12):e81704. doi: 10.1371/journal.pone.0081704 (PMC3847036; doi:10.1371/journal.pone.0081704)
Supplement: Table S8 — Mean census size and analysis of molecular variance (AMOVA) within Alpine linguistic groups under study based on 15 Y chromosome STRs (acronyms as in Table 1). (DOC) [file pone.0081704.s013.doc]

**Supplementary Table S8.** Mean census size and Analysis of molecular variance (AMOVA) within Alpine linguistic groups based on 15 Y chromosome STRs (acronyms as in Table 1).

| **Group** | **Populations** | **Mean census size** | **Within group diversity** | **p value** |
| --- | --- | --- | --- | --- |
| German speakers | LES-LUS-SAP-SAU-TIM | 3195 | 0.240 | 0.000 |
| *Linguistic islands* | SAP-SAU-TIM | 745 | 0.156 | 0.000 |
| *Cimbrians* | LES-LUS | 6870 | 0.387 | 0.000 |
| Italian speakers | ADI-FER-FIE-GIU-NON-PRI-SOL | 41038 | 0.040 | 0.000 |
| *Eastern Trentino* | FER-FIE-PRI | 10508 | 0.075 | 0.000 |
| *Western Trentino* | GIU-NON-SOL | 29783 | 0.028 | 0.000 |
| Ladin speakers | BAD-FAS-GAR | 10245 | 0.075 | 0.000 |
