# Supplementary material for: Demographic Histories, Isolation and Social Factors as Determinants of the Genetic Structure of Alpine Linguistic Groups
Source: PLoS One. 2013 Dec 2;8(12):e81704. doi: 10.1371/journal.pone.0081704 (PMC3847036; doi:10.1371/journal.pone.0081704)
Supplement: Table S9 — Analysis of molecular variance (AMOVA) within groups under study based on 5 Y chromosome STRs, including results of jacknife procedure (acronyms as in Table 1, Table S1 and S4). (DOC) [file pone.0081704.s014.doc]

**Supplementary Table S9.** Within group diversity among population groups under study based on 5 Y chromosome STRs (DYS19, 390, 391, 392 and 393) and the hypervariable region of mtDNA (from 16033 to 16365), including the results of jacknife procedure (acronyms as in Table 1, Supplementary Table S1 and S4).

|  |  | **Y chromosome** | | **mtDNA** | |
| --- | --- | --- | --- | --- | --- |
| **Group** | **Populations** | **Within group** | **p value** | **Within group** | **p value** |
| German speakers | LES-LUS-SAP-SAU-TIM | 0.315 | 0.000 | 0.077 | 0.000 |
|  | Excluding LES | 0.380 | 0.000 | 0.091 | 0.000 |
|  | Excluding LUS | 0.184 | 0.000 | 0.086 | 0.000 |
|  | Excluding SAP | 0.314 | 0.000 | 0.057 | 0.000 |
|  | Excluding SAU | 0.371 | 0.000 | 0.081 | 0.000 |
|  | Excluding TIM | 0.315 | 0.000 | 0.065 | 0.000 |
| Italian speakers | ADI-FER-FIE-GIU-NON-PRI-SOL | 0.053 | 0.000 | 0.008 | 0.010 |
|  | Excluding ADI | 0.061 | 0.000 | 0.010 | 0.004 |
|  | Excluding FER | 0.055 | 0.000 | 0.008 | 0.012 |
|  | Excluding FIE | 0.053 | 0.000 | 0.009 | 0.016 |
|  | Excluding GIU | 0.056 | 0.000 | 0.009 | 0.014 |
|  | Excluding NON | 0.061 | 0.000 | 0.009 | 0.005 |
|  | Excluding PRI | 0.023 | 0.001 | 0.009 | 0.009 |
|  | Excluding SOL | 0.066 | 0.000 | 0.006 | 0.053 |
| Ladin speakers | BAD-FAS-GAR | 0.077 | 0.001 | 0.035 | 0.000 |
| North-Eastern Italy* | BRE-TRE-VIC | -0.003 | 0.555 | 0.001 | 0.360 |
| Pyrenean | ARA-CER-CIN-JAC-URG | 0.018 | 0.026 | n.a. | n.a. |
|  | Excluding ARA | 0.011 | 0.100 | n.a. | n.a. |
|  | Excluding CER | 0.031 | 0.009 | n.a. | n.a. |
|  | Excluding CIN | 0.002 | 0.339 | n.a. | n.a. |
|  | Excluding JAC | 0.025 | 0.015 | n.a. | n.a. |
|  | Excluding URG | 0.024 | 0.024 | n.a. | n.a. |
| South Tyroleans | ISA-PUS-VEL-VEU | 0.004 | 0.265 | 0.030 | 0.000 |
|  | Excluding ISA | 0.004 | 0.293 | 0.014 | 0.016 |
|  | Excluding PUS | 0.007 | 0.187 | 0.033 | 0.000 |
|  | Excluding VEL | 0.011 | 0.131 | 0.030 | 0.001 |
|  | Excluding VEU | -0.006 | 0.704 | 0.046 | 0.000 |

* This group is composed by three geographically close plain populations (see Supplementary Table 4).
